# Allergic rhinitis as a potentially modifiable contributor to small airway dysfunction and incomplete clinical remission in severe asthma^☆^

**DOI:** 10.1016/j.waojou.2026.101484

**Published:** 2026-10-28

**Authors:** Huan Wu, Qin Ouyang

**Affiliations:** Zhejiang Chinese Medical University Affiliated Wenzhou Hospital of Traditional Chinese Medicine, Wenzhou, Zhejiang, China

To the Editors,

We read with great interest the article by Al-Ahmad et al, which demonstrated that small airway dysfunction (SAD) is a functional barrier to spirometry-defined clinical remission (CR) in severe asthma treated with biologic therapies.^1^ We congratulate the authors on this important work, which highlights the complementary value of impulse oscillometry (IOS) and spirometry in assessing distal airway impairment.

However, we would like to draw attention to one finding that deserves further discussion: the prevalence of allergic rhinitis (AR) was significantly higher in patients with SAD than in those without SAD (93.1% vs. 73.3%, P = 0.003).^1^ This observation strongly supports the concept of “united airway disease,” in which the upper and lower airways share anatomical, functional, and immunological connections, and Th2-driven inflammatory mechanisms affect both compartments.^2^ AR in severe asthma may therefore be more than a mere comorbidity — it may be a potentially modifiable contributor to persistent small airway dysfunction.

Several pathophysiological mechanisms may link AR to SAD. First, postnasal drip can deliver inflammatory cells and pro-inflammatory mediators directly to the lower airways. Second, nasobronchial reflexes mean that nasal inflammation can influence bronchial tone through neural pathways. Third, nasal obstruction leads to oral breathing, exposing the lower airways to unfiltered, unheated, and unhumidified air, increasing the deposition of allergens and irritants. Furthermore, uncontrolled nasal inflammation may sustain systemic type 2 inflammation, and peripheral airway dysfunction may persist even when biologics effectively suppress lower airway inflammation.^3^

Importantly, evidence suggests that small airway function is already abnormal in patients with AR even in the absence of asthma symptoms. A cross-sectional case-control study using IOS found that non-asthmatic children with moderate-to-severe persistent AR exhibited significantly higher R5 and R5-R20 values compared with healthy controls, indicating subclinical small airway dysfunction despite normal spirometry.^4^ Another study in adolescents with AR demonstrated that airway resistance was elevated compared with healthy controls, and this elevation was correlated with nasal oxidative stress and endothelial damage, independent of asthma status.^5^ A 2025 study further showed that small airway impairment was present in patients with house dust mite-allergic rhinitis, with 67.3% of AR children with abnormal pulmonary ventilation exhibiting small airway dysfunction.^6^ These findings support the hypothesis that SAD may be an intermediate step in the progression from AR to asthma.

Appropriate treatment of AR may improve lower airway function. A randomized, double-blind, placebo-controlled crossover study demonstrated that intranasal beclomethasone dipropionate significantly improved methacholine PC20 in patients with perennial AR and mild asthma after 4 weeks of treatment, confirming that treatment of upper airway inflammation can indirectly improve bronchial hyperreactivity.^7^ A randomized study showed that pranlukast, a leukotriene receptor antagonist, improved allergic rhinitis symptoms, asthma symptoms, and pulmonary function after 3 months of therapy in patients with asthma-associated AR.^8^ A study in children with AR and SAD showed that combination therapy significantly improved lung function and reduced inflammation.^9^ These findings suggest that AR treatment may not only improve nasal symptoms but also be associated with beneficial effects on distal airway function.

However, Al-Ahmad et al. did not report AR severity, duration, treatment, or control status.^1^ Important variables such as nasal corticosteroid use, antihistamines, leukotriene receptor antagonists, allergen immunotherapy, and treatment adherence were not included in the analysis. These factors could influence lower airway inflammation and small airway function, and may therefore modify the likelihood of achieving CR. Without these data, it remains difficult to determine whether AR is simply a marker of more severe type 2 inflammation or a potentially modifiable contributor to persistent SAD.

It is also important to note that the cross-sectional design of the study cannot establish causality.^1^ The association between AR and SAD observed at baseline may reflect shared underlying inflammatory pathways rather than a direct causal relationship. Prospective longitudinal studies are needed to determine whether AR precedes and contributes to the development or persistence of SAD.

Interestingly, the authors observed fewer exacerbations and less OCS use in some SAD subgroups (68.97% vs. 50.67%, P = 0.03 for combined modalities; 65.17% vs. 45.45%, P = 0.03 for IOS),^1^ which may reflect phenotypic heterogeneity, as SAD often represents a subclinical “silent zone” impairment that does not immediately manifest as exacerbation-prone disease.^10^ Alternatively, patients with AR may seek medical attention earlier and use intranasal corticosteroids more frequently, thereby indirectly improving some asthma outcomes despite persistent functional impairment. This possibility is particularly relevant because it would support the hypothesis that AR is a modifiable contributor to asthma outcomes. Nevertheless, the persistent association between AR and SAD warrants further investigation, particularly regarding whether AR control status modifies this relationship.

We therefore suggest 2 priorities for future research that balance research ideals with practical feasibility in severe asthma cohorts: (1) incorporate validated, easily obtainable measures of AR severity and control, such as the Total Nasal Symptom Score (TNSS) and nasal nitric oxide, and record AR treatment intensity including nasal corticosteroid dosage, antihistamine use, leukotriene receptor antagonists, and immunotherapy;^11^, ^12^ and (2) stratify patients by AR control status in both retrospective and prospective analyses to determine whether optimal AR management is associated with improved small airway function and a higher likelihood of clinical remission.^13^ Where feasible, nasal endoscopy and nasal eosinophil counts could provide additional mechanistic insights.

The authors found that CR was achieved in only 17.3% of patients and was significantly less frequent among those with SAD (8.2–8.6% vs. 28.3%).^1^ Inadequate AR treatment may have contributed to this residual functional impairment, and this possibility warrants further investigation.

We agree that SAD is an important functional barrier to clinical remission in severe asthma.^10^, ^12^, ^13^ We believe that AR should be considered not merely a comorbidity but a potentially treatable contributor to persistent small airway dysfunction. We recommend that comprehensive upper airway assessment be integrated into both research and clinical management of severe asthma, and that future prospective studies explore whether integrated upper and lower airway management can improve remission outcomes under biologic therapy.

We hope these comments will stimulate further investigation into the upper airway as a potentially modifiable contributor to SAD and incomplete clinical remission.

## Declaration of generative AI and AI-assisted technologies in the writing process

During the preparation of this work, the authors used **DeepL and DeepSeek** in order to **translate and polish the language of the manuscript**. After using these tools, the authors reviewed and edited the content as needed and take full responsibility for the content of the publication.

## Funding

None.

## Conflict of interest

The authors declare no conflict of interest.
